# Robust Output Feedback Control of Single-Link Flexible-Joint Robot Manipulator with Matched Disturbances Using High Gain Observer

**DOI:** 10.3390/s21093252

**Published:** 2021-05-08

**Authors:** Hameed Ullah, Fahad Mumtaz Malik, Abid Raza, Naveed Mazhar, Rameez Khan, Anjum Saeed, Irfan Ahmad

**Affiliations:** Department of Electrical Engineering, CEME, National University of Sciences and Technology, Islamabad 44000, Pakistan; malikfahadmumtaz@ceme.nust.edu.pk (F.M.M.); abid.raza@ceme.nust.edu.pk (A.R.); naveed.mazhar@ceme.nust.edu.pk (N.M.); rameez.khan@ceme.nust.edu.pk (R.K.); anjum.saeed16@ee.ceme.edu.pk (A.S.); iahmad17@ee.ceme.edu.pk (I.A.)

**Keywords:** flexible-joint robotic manipulator, high-gain observers, output feedback control, robust control, sliding mode control

## Abstract

This article focuses on the output feedback control of single-link flexible-joint robot manipulators (SFJRMs) with matched disturbances and parametric uncertainties. Formally, four sensing elements are required to design the controller for single-link manipulators. We have designed a robust control technique for the semiglobal stabilization problem of the angular position of the link in the SFJRM system, with the availability of only a position sensing device. The sliding mode control (SMC) based output feedback controller is devised for SFJRM dynamics. The nonlinear model of SFJRM is considered to estimate the unknown states utilizing the high-gain observer (HGO). It is shown that the output under SMC using HGO-based estimated states coincides with that using original states when the gains of HGO are sufficiently high. Finally, the results are presented showing that the designed control technique works well when the SFJRM model is uncertain and matched perturbations are expected.

## 1. Introduction

For the past several years, there has been extensive on-going research on control of flexible joint robotic manipulators (FJRMs). This is because advanced robotic applications require light-weight robots which could be driven by utilizing less quantity energy. In the modern era, cost-effective robust solutions to engineering problems are highly focused. Robotic manipulator is one of the fundamental parts of many industrial, medical, and agricultural applications. The robotic manipulator is a complex nonlinear system that has been widely exploited in a multitude of industries, for example, the beverage factories, and car-assembly plants, space [1], underwater vehicles [2], agriculture [3], automation [4], and many more. In several industrial applications, the industrial robot’s stiffness properties are very important. The authors in [5], developed an industrial robot’s compliant joint dynamic model, in which an impulsive modal analysis approach is used to experimentally identify the joint stiffness. In addition to industrial applications, it has also been extensively applied in the medical field to manipulate objects and to interact with the dynamic environment [6]. Moreover, it is also being used worldwide in the operating room to reduce the hospital time, cost, patient discomfort, and to improve the surgical procedure by bringing precision and the capability to access surgical areas with miniaturized instruments remotely [7].

To compare the conventional heavy-weight and rigid-link robotic manipulators (RRMs), FJRMs have inherent advantages over the RRMs such as lightweight, smaller dimensions, better maneuverability, better transportability, lower power consumptions, less control effort, large work volume, lower cost, fast motion, safer operations, smaller actuators, and higher operational speed due to reduced inertia [8,9,10,11,12,13]. With the widespread applications and rapid development of robotic technology, under different types of environments, the requirement for well satisfactory working and flexible control is becoming increasingly demanding.

For designing an efficient and robust control scheme, the essential and primary step is to calculate an accurate dynamic model of FJRM system. The degree of freedom (DOF) of the FJRM systems is greater than its number of actuators which means that’s it is an underactuated system [14]. For FJRMs, design of controller is intrinsically more complicated as no exclusive control input be present for each DOF independently [15]. Majority of controllers designed for industrial robots are based on rigid-link assumption [16]. To take into account the joint flexibility, for n-link robots, it requires 2n generalized coordinates which define its entire dynamic behavior [17]. Therefore, due to flexibility in joint, the dynamical modelling becomes more complex compared to that of a rigid-link robotic manipulator. Though mathematical modelling is merely the real system’s approximation, therefore system behavior’s simplified representations certainly contain of modelling inaccuracies like parametric and modelling uncertainties, vibrations, and external disturbances. Modelling inaccuracies, chaotic phenomenon, friction, vibrations, extremely uncertain working conditions, inherently high nonlinearities, and change of payload make the controller design challenging [16,18]. In industrial and space applications, we require a controller that is capable of overcome modelling uncertainties and disturbance effects.

To address the aforementioned problems, several engineers and researchers have investigated numerous linear and nonlinear control design topologies for the FJRMs system. For stabilization of flexible-joint robots, proportional integral derivative (PID) controller has been designed by many researchers as being classical and simplest control technique [19,20,21,22]. To address trajectory tracking control problems, the authors in [23] designed dynamic feedback control for FJRMs. The work of [23] assumes that the measurement of angular positions of link and motor are available, whereas the desired velocities in controller are estimated by reduced-order observer. A similar approach is used in [24], in which velocity observer is implemented based on a singular perturbation approach where the controller needs sensors for position measurements and elastic force. In another work, the linear matrix inequality techniques were suggested in [25] for the robust observer design and observer-based controller. For FJRMs position control, Tomei in [26] used a simple proportional derivative (PD) control in which a full state measurement was required. The finite-time state feedback controllers are proposed for robotic manipulators by the authors in [27], which guarantees the state convergence for case of both bounded and unbounded control signals. Hu et al. in [28], proposed the output feedback control (OFC) procedures, which have incited rising attention in the tracking control area at current time and brings the feasible routes into designing closed-loop tracking controller for FJRMs system with position sensing only. In [29], the authors designed an adaptive controller to guarantee a high precision position regulation of the flexible joint robots under uncertainties. For a class of FJRM system, by using state-dependent Riccati equations, a finite-time optimal controller was proposed by authors in [30]. Furthermore, by using the full states to sustain the tracking ability, authors in [31] investigated an industrial flexible joint. In [32] an adaptive control method was proposed, without the information of the angular acceleration, the parameter identification techniques were implemented for the flexible-joint robotic systems. To enhance the robustness and guarantee the stability of a class of FJRMs system, authors in [33] designed full state-feedback neural network control. The performance of most of the control strategies described above is appropriate for nominal system, however, to deal with the unmodeled dynamic uncertainties, parameter perturbations, faults and external disturbances is still a challenge.

Some active disturbance rejection control techniques are suggested for robotic manipulator systems to compensate and actively estimate the disturbance [34,35,36]. To address the effect of mismatched disturbances, authors in [37] proposed a backstepping-based approach in conjunction with disturbance observer by using the disturbance rejection method for nonlinear systems. In [38] a generalized momentum based finite time disturbance observer is proposed for robotic manipulators with assumption that sensors for all states are available. For uncertain FJRM system motion control, a robust control technique for trajectory tracking based-on the extended-state-observers-based controller was proposed in [39]. However, with both external disturbances and parametric perturbations for the FJRM system, the performance of this method was not acceptable for advanced applications. Furthermore, multiple sensors are needed in these methods, which will not only bring additional noise but also affect joint flexibility. Most of the work presented assumes all states variable availability, thus robustness somewhere guaranteed are depending on modelling.

Generally, control laws via a feedback control need availability of all the states variable, i.e., link positions, acceleration, jerk, and velocity. However, the position might be precisely measured, noise disturbs velocity of the joint. Furthermore, by using numerical differentiation in noisy measurement may lead to difficulties to obtain the unmeasured states. Note that at least measurement of one state using sensor or knowledge of initial conditions of the system is compulsory to design an observer. In practical scenario, it is difficult to measurement all the state variables, or even sometimes not feasible, because of technical or economic reasons as sensors are needed for each state of the systems [40,41]. To address this drawback, OFC can be designed that measures output of the systems whereas for estimation of unknown remaining states an observer is used. In linear system case, states can be estimated by using linear observers, however, the state estimation of the complex nonlinear system is a challenging task and has gained vast consideration in literature [42,43] and the references therein. Moreover, the traditional nonlinear sensorless state estimators like sliding mode observer, backstepping observer, Kalman observer, etc. can be designed only as part of the controller, and hence not only the complexity of design increases but also the reusability of estimated states (with other control technique) is not possible. To overcome this challenge, high-gain observer (HGO) is one of the most useful and powerful techniques to be used for nonlinear OFC.

In the past several years, HGO has been considered as the essential technique used to design OFC of the nonlinear systems and to estimate their unmeasured states [10]. HGO has played an important part in advancement of regulation theory for nonlinear systems. Furthermore, in presence of model uncertainties, HGO is robust and has capability to estimate states of nonlinear systems, presented in normal form [44]. One of the most important properties of HGO is the separation principle. The combination of the globally bounded state feedback controller (SFC) and HGO allow for the separation approach. First, the SFC is designed that stabilizes the systems and meet the requirements. Secondly, the OFC is obtained by replacing the original states with its estimated states, provided by HGO [44,45]. It is essential to affirm that the separation principle is a unique feature in the HGO case which does not happen in other separation-principle results, including linear systems, and that is state trajectories recovery by making the observers sufficiently fast. For a wide class of nonlinear systems, HGO is used and guarantees that for sufficiently high gain of the observer the OFC recovers the performance of SFC.

In this article, robust sliding mode control (SMC) technique is designed in conjunction with a high-gain observer to overcome these challenges. Owing to its outstanding robust nature and computational simplicity, SMC has attained popularity in several scientific applications [46,47]. To deal with bounded external disturbances, perturbations, and uncertainties of nonlinear systems, SMC is one of the most widely used powerful methods. This is because of its fast convergence, strong robustness against perturbations, parameter variations, external disturbances, and model uncertainties [48,49,50,51].

It is notable from the aforementioned study, that there is no significant work for output feedback control of SFJRM using nonlinear dynamics. In the work of [39], the linear observer is proposed to resolve the same problem but the performance of the proposed output feedback controller is valid only locally. In this article, a control solution is proposed for semiglobal stabilization problem of the angular position of the link in SFJRM system with the availability of only a position sensing device. It is theoretically proved and validated in simulations that knowledge of exact parametric values is not required to achieve the same controller performance as in presence of a sensor for each state. Furthermore, the angular rate of the actuating motor is assumed to be distorted by unknown bounded disturbance. The conventional SMC is used in conjunction with HGO to suppress the effects of this distortion upon the systems.

The rest of the article is planned as; Section 2 describes the dynamical modelling of a SFJRM and problem formulation. In Section 3, SMC for the SFJRM is designed, followed by HGO design which is introduced in Section 4. MATLAB/Simulink (MathWorks Inc., Natick, MA, USA) results and discussion are presented in Section 5. Conclusion is presented in the last section.

## 2. Dynamical Model and Problem Statement

In this section, the mathematical modelling of SFJRM is explained. The working of the system is demonstrated in detail. Finally, the problem statement of this article is given along with basic technical definitions.

### 2.1. Dynamical Model of SFJRM

**Figure 1:**
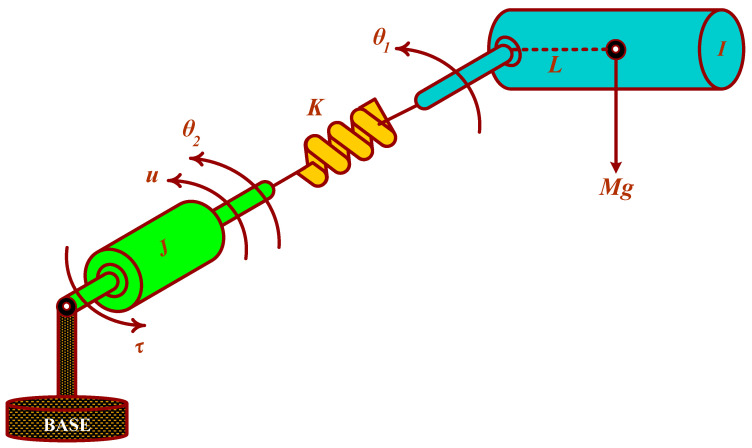
SFJRM schematic diagram.

The basic schematic diagram of the SFJRM is shown in Figure 1. Its nonlinear dynamical model can be written as [39]:

(1) $$I{\ddot{\theta }}_{1}+MgL\sin\theta_{1}+K\left(\theta_{1}-\theta_{2}\right)=0$$

(2) $$J{\ddot{\theta }}_{2}-K\left(\theta_{1}-\theta_{2}\right)=\tau$$

where $\theta_{1}$ and $\theta_{2}$ are the angular positions of the link and actuator, respectively, I and J are the inertias of link and actuator respectively, M is the link-mass, g is the gravitational constant, L is the distance of the mass from the center, K denotes the stiffness of linear spring, τ is the input torque applied to the actuator shaft while the viscous damping has been neglected [16,39]. For simplification, the nonlinear dynamical model of the SFJRM (1)–(2) can be denoted in state-space form. Defining $z_{1}=\theta_{1}$, $z_{2}={\dot{\theta }}_{1}$, $z_{3}=\theta_{2}$, $z_{4}={\dot{\theta }}_{2}$ and u=τ. Then, the system (1)–(2) takes the form:

(3) $${\dot{z}}_{1}=z_{2}$$

(4) $${\dot{z}}_{2}=-\frac{MgL}{I}\sin z_{1}-\frac{K}{I}\left(z_{1}-z_{3}\right)$$

(5) $${\dot{z}}_{3}=z_{4}$$

(6) $${\dot{z}}_{4}=\frac{K}{J}\left(z_{1}-z_{3}\right)+\frac{u\;}{J}$$

Since it is desired to stabilize the angular position of the link, hence the output of the system can be defined by:

(7) $$y=h\left(z\right)=z_{1}$$

### 2.2. Problem Statement and Preliminaries

Design a controller for stabilization of angular position of the link in SFJRM system (3)–(6) under that the following limitations:

(i) Sensing device is available only to measure the output i.e., position of SFJRM
(ii) The parametric values of the system (K, τ, and M) are not exactly known
(iii) The angular rate of the actuator is subjected to unknown bounded disturbances.

**Remark** **1.** *In the context of control systems, the goal is to design a robust OFC such that the effect of parametric uncertainties and matched perturbations is diminished.*

**Definition** **1.** *References* ([52,53]) *a system is said to be in singularity perturbed form if its dynamics can be represented as:*

(8) $$\dot{x}=𝒻\left(t,x,z,u,\epsilon \right)$$

(9) $$\epsilon \dot{z}=ℊ\left(t,x,z,u,\epsilon \right)$$

*where* 𝒻 *and* ℊ *are continuously differentiable vector fields,* $\epsilon \in \left(0,1\right)$ *is singular perturbation parameter and satisfies* ϵ≪1. *The state vectors are defined by* $x\in D_{x}\subset {\mathbb{R}}^{m}$ *and* $z\in D_{z}\subset {\mathbb{R}}^{n}$, *while* $u\in D_{u}\subset {\mathbb{R}}^{p}$ *denotes the input vector. Moreover, the states x and z are called slow and fast states, respectively*.

**Definition** **2.** *Reference* ([54]) *a single-input single-output (SISO) nonlinear system* $\dot{\xi }=f\left(\xi \right)+g\left(\xi \right)u$ *has a relative degree* r *if*

(i) $L_{g}L_{f}^{\rho }h\left(\xi \right)=0\;\forall \rho <r-1$ *and for* ∀ ξ *in the neighborhood of* $\xi^{o}.$
(ii) $L_{g}L_{f}^{r-1}h\left(\xi^{o}\right)\neq 0$.

*where* f *and* g *are continuously differential vector fields,* $\xi^{o}$ *denotes the equilibria of* ξ *and*

(10) $$L_{f}^{\rho }h\left(\xi \right)=\frac{\partial \left(L_{f}^{\rho -1}h\right)}{\partial \xi }f\left(\xi \right)$$

Furthermore, $L_{f}h\left(\xi \right)=\frac{\partial h}{\partial \xi }f\left(\xi \right)$. The Lie derivatives of the system are given according to definition 2 as; $L_{g}h\left(z\right)=0$, $L_{f}h\left(z\right)=z_{2}$, $L_{g}L_{f}h\left(z\right)=0$, $L_{f}^{2}h\left(z\right)=z_{3}$, $L_{g}L_{f}^{2}h\left(z\right)=0$, $L_{f}^{3}h\left(z\right)=z_{4}$, $L_{g}L_{f}^{3}h\left(z\right)=K/IJ$. Since, K, I, and J are non-zero, therefore, $L_{g}L_{f}^{3}h\left(z\right)\neq 0$. So, the system’s relative degree r can be calculated as:

$$L_{g}L_{f}^{r-1}h\left(z^{o}\right)=L_{g}L_{f}^{3}h\left(z\right)$$

By comparing we get; r−1=3⇒ r=4 ∀ z∈R and K/IJ≠0. The system’s relative degree r is equal to the order of the system i.e., n=r=4, which indicates that the system has no zero-dynamics and hence, the system dynamical model is completely linearizable through feedback.

## 3. Sliding Mode Control Design

SMC is one of the commonly used robust control techniques for a wide class of uncertain nonlinear systems. The design procedure consists of two main steps:

1. Design of sliding surface
2. Design of a discontinuous control to establish the sliding mode

Sliding mode control technique is advantageous because of its invariance to bounded matched uncertainties, finite-time convergence to the sliding surface, and reduced order of sliding equation. However, with these advantages, sliding mode control has some disadvantages for example chattering, unable to tackle mismatched uncertainty, and asymptotic convergence of state variables.

Note that a nonlinear system can be transformed, utilizing an appropriate change of coordinates in the state space, into the “normal form” of special interest, on which numerous significant properties can be elucidated [54]. The nonlinear dynamic system (3)–(7) is not in normal form. To simplify the control design, we will use a nonlinear coordinate transformation so that the system can be represented in normal form. By applying the nonlinear coordinate transformation of the form $\xi =T\left(z\right)$, the original dynamics (3)–(7) can be re-written in terms of the transformed new coordinates as [39]:

(11) $$T\left(z\right)=\left[\begin{array}{c} h\left(z\right)\\ L_{f}h\left(z\right)\\ L_{f}^{2}h\left(z\right)\\ L_{f}^{3}h\left(z\right) \end{array}\right]$$

where $L_{f}h\left(z\right)=z_{2}$, $L_{f}^{2}h\left(z\right)=-\frac{MgL}{I}\sin z_{1}-\frac{K}{I}\left(z_{1}-z_{3}\right),$ and $L_{f}^{3}h\left(z\right)=-\frac{MgL}{I}\cos\left(z_{1}\right)z_{2}-\frac{K}{I}\left(z_{2}-z_{4}\right)$. Moreover, the transformation is global transformation since the relative degree of the system is defined for all ξ∈ℝ, thus by the inverse function theorem, the inverse transformation is also defined for all z∈ℝ. Then the new coordinates are given by:

(12) $$\xi_{1}=z_{1}$$

(13) $$\xi_{2}=z_{2}$$

(14) $$\xi_{3}=-\frac{MgL}{I}\sin z_{1}-\frac{K}{I}\left(z_{1}-z_{3}\right)$$

(15) $$\xi_{4}=-\frac{MgL}{I}\cos\left(z_{1}\right)z_{2}-\frac{K}{I}\left(z_{2}-z_{4}\right)$$

**Remark** **2.** *Since the transformed coordinates are themselves physically meaningful as can be seen that* $\xi_{1}$ *,* $\xi_{2}$ *,* $\xi_{3}$ *and* $\xi_{4}$ *are the link position, velocity, acceleration, and jerk respectively. As the system model is defined in these coordinates after transformation, thus these are the natural variable to use for control.*

The normal form of the dynamical system which is in new coordinates is represented as:

(16) $${\dot{\xi }}_{1}=\xi_{2}$$

(17) $${\dot{\xi }}_{2}=\xi_{3}$$

(18) $${\dot{\xi }}_{3}=\xi_{4}$$

(19) $${\dot{\xi }}_{4}=F\left(\xi \right)+bu$$

(20) $$y=h\left(\xi \right)=\xi_{1}$$

where $b=\frac{K}{IJ}$ and

(21) $$F\left(\xi \right)=-\frac{MgL}{I}\sin\xi_{1}\left(\frac{K}{J}-\xi_{2}^{2}\right)-\left(\frac{K}{I}+\frac{K}{J}+\frac{MgL}{I}\cos\xi_{1}\right)\xi_{3}$$

Re-writing the Equations (16)–(20) in generalized form:

(22) $$\dot{\xi }=A\xi +B\phi \left(\xi ,u\right)$$

(23) y=Cξ

where $\xi \in {\mathbb{R}}^{4}:\xi ={\left[\begin{array}{cccc} \xi_{1} & \xi_{2} & \xi_{3} & \xi_{4} \end{array}\right]}^{T}$, A is 4×4 matrix, B is 4×1*,* C is 1×4*,* and $\phi :{\mathbb{R}}^{4}\times \mathbb{R}\to \mathbb{R}$ is a real-valued map, and $\phi \left(\xi ,u\right)$ is the image of $\left(\xi ,u\right)$ under the map given by:

$$A=\left[\begin{array}{cccc} 0 & 1 & 0 & 0\\ 0 & 0 & 1 & 0\\ 0 & 0 & 0 & 1\\ 0 & 0 & 0 & 0 \end{array}\right],\;\;B=\left[\begin{array}{c} 0\\ 0\\ 0\\ 1 \end{array}\right],\;\;C=\left[\begin{array}{cccc} 1 & 0 & 0 & 0 \end{array}\right],$$

And $\phi \left(\xi ,u\right)\;=\;F\left(\xi \right)\;+\;bu\;+\;\Upsilon \left(t\right)$, where $\Upsilon \left(t\right)$ is the matched uncertain term introduced in the system due to external disturbances.

Assumption-1: There exists some positive constant L such that the uncertain function satisfies

(24) $$\left|\Upsilon \left(t\right)\right|\leq L$$

**Remark** **3.** *We assume that in the system (16)–(20), the function *$F\left(\xi \right)$*is the uncertain function due to parametric variations because of external effects and uncertainties in measuring these parameters. Thus, we know only the upper bound of an uncertain function*.

We consider the sliding surface s such that

(25) $$s=c_{1}\xi_{1}+c_{2}\xi_{2}+c_{3}\xi_{3}+\xi_{4}$$

where $c_{1},\ldots ,c_{3}$ are chosen such that the polynomial $s^{3}+c_{1}s^{2}+c_{2}s+c_{3}=0$ is Hurwitz.

Consider the Lyapunov function candidate

(26) $$V\left(s\right)=\frac{1}{2}s^{2}$$

Taking the time derivative of $V\left(s\right)$

(27) $$\dot{V}\left(s\right)=s\dot{s}=s\left(c_{1}\xi_{2}+c_{2}\xi_{3}+c_{3}\xi_{4}+F\left(\xi \right)+\Upsilon \left(t\right)+bu\right)$$

Let us consider the control input

(28) $$u=\left(-c_{1}\xi_{2}-c_{2}\xi_{3}-c_{3}\xi_{4}-F\left(\xi \right)-\beta \mathrm{sgn}\left(s\right)\right)/b$$

where β is the design parameter, positive constant and sgn is the signum function given by:

(29) $$sgn\left(s\right)=\left\{\begin{array}{cc} 1, & s>0\\ 0, & s=0\\ -1, & s<0 \end{array}\right.$$

Substituting (28) into (27), we get:

(30) $$\dot{V}\left(s\right)=s\left(\Upsilon \left(t\right)-\beta \mathrm{sgn}\left(s\right)\right)$$

(31) $$\dot{V}\left(s\right)\leq s\left(L-\beta \mathrm{sgn}\left(s\right)\right)$$

(32) $$\dot{V}\left(s\right)\leq \left|s\right|L-\beta \left|s\right|$$

(33) $$\dot{V}\left(s\right)\leq -\left|s\right|\left(\beta -L\right)$$

Taking β=L+K

(34) $$\dot{V}\left(s\right)\leq -K\left|s\right|$$

Thus $\dot{V}\left(s\right)$ is negative definite, which implies that the states reach the sliding manifold in finite-time and stabilize to the origin independent of the uncertain function $\Upsilon \left(t\right)$, and hence ensuring the robustness property of the SMC.

**Remark** **4.** *The SMC derived in this section considers signum function as a discontinuous control law that not only introduces chattering in the control input but also makes the control law non-Lipchitz. We will use an approximation of signum function by replacing it with saturation function in control law and by abuse of notation will still call it SMC*.

## 4. High-Gain Observer Design

For the FJSRM, the only state $\xi_{1}$ is known. The following HGO is proposed that uses the only available state $\xi_{1}$ which is the measured output of the system:

(35) $${\dot{\hat{\xi }}}_{1}={\hat{\xi }}_{2}+\hbar_{1}\left(\xi_{1}-{\hat{\xi }}_{1}\right)$$

(36) $${\dot{\hat{\xi }}}_{2}={\hat{\xi }}_{3}+\hbar_{2}\left(\xi_{1}-{\hat{\xi }}_{1}\right)$$

(37) $${\dot{\hat{\xi }}}_{3}={\hat{\xi }}_{4}+\hbar_{3}\left(\xi_{1}-{\hat{\xi }}_{1}\right)$$

(38) $${\dot{\hat{\xi }}}_{4}=F\left(\hat{\xi }\right)+bu+\hbar_{4}\left(\xi_{1}-{\hat{\xi }}_{1}\right)$$

where $\hbar_{1}=\alpha_{1}/\varepsilon ,\;\hbar_{2}=\alpha_{2}/\varepsilon^{2},\;\hbar_{3}=\alpha_{3}/\varepsilon^{3}$ and $\hbar_{4}=\alpha_{4}/\varepsilon^{4}$. Generally, we can write as;

(39) $$\dot{\hat{\xi }}=A\hat{\xi }+B\phi_{0}\left(\hat{\xi },u\right)+H\left(y-C\hat{\xi }\right)$$

where, $\phi_{0}\left(\hat{\xi },u\right)=F\left(\hat{\xi }\right)+bu$, is the nominal model of $\phi =\left(\xi ,u\right)$ and observer gain is defined as;

$$H={\left[\begin{array}{cccc} \hbar_{1} & \hbar_{2} & \hbar_{3} & \hbar_{4} \end{array}\right]}^{T}$$

and the constant $a_{i}$’s are chosen such that the polynomial

$$s^{4}+\alpha_{1}s^{3}+\alpha_{2}s^{2}+\alpha_{3}s+\alpha_{4}=0$$

is Hurwitz, and 0<ε<1 is the small positive constant also called the high-gain parameter.

Convergence Analysis:

The estimation error of the observer can be represented as:

(40) $$\tilde{\xi }=\left[\begin{array}{c} {\tilde{\xi }}_{1}\\ {\tilde{\xi }}_{2}\\ {\tilde{\xi }}_{3}\\ {\tilde{\xi }}_{4} \end{array}\right]=\left[\begin{array}{c} \xi_{1}-{\hat{\xi }}_{1}\\ \xi_{2}-{\hat{\xi }}_{2}\\ \xi_{3}-{\hat{\xi }}_{3}\\ \xi_{4}-{\hat{\xi }}_{4} \end{array}\right]$$

Taking the derivative of (40) and substituting (16)–(19) and (35)–(38) we obtain as:

(41) $$\left[\begin{array}{c} {\dot{\tilde{\xi }}}_{1}\\ {\dot{\tilde{\xi }}}_{2}\\ {\dot{\tilde{\xi }}}_{3}\\ {\dot{\tilde{\xi }}}_{4} \end{array}\right]=\left[\begin{array}{c} {\dot{\xi }}_{1}-{\dot{\hat{\xi }}}_{1}\\ {\dot{\xi }}_{2}-{\dot{\hat{\xi }}}_{2}\\ {\dot{\xi }}_{3}-{\dot{\hat{\xi }}}_{3}\\ {\dot{\xi }}_{4}-{\dot{\hat{\xi }}}_{4} \end{array}\right]=\left[\begin{array}{c} \xi_{2}-{\hat{\xi }}_{2}-\hbar_{1}\left(\xi_{1}-{\hat{\xi }}_{1}\right)\\ \xi_{3}-{\hat{\xi }}_{3}-\hbar_{2}\left(\xi_{1}-{\hat{\xi }}_{1}\right)\\ \xi_{4}-{\hat{\xi }}_{4}-\hbar_{3}\left(\xi_{1}-{\hat{\xi }}_{1}\right)\\ F\left(\xi \right)-F\left(\hat{\xi }\right)-\hbar_{4}\left(\xi_{1}-{\hat{\xi }}_{1}\right) \end{array}\right]=\left[\begin{array}{c} {\tilde{\xi }}_{2}-\frac{\alpha_{1}}{\varepsilon }{\tilde{\xi }}_{1}\\ {\tilde{\xi }}_{3}-\frac{\alpha_{2}}{\varepsilon^{2}}{\tilde{\xi }}_{1}\\ {\tilde{\xi }}_{4}-\frac{\alpha_{3}}{\varepsilon^{3}}{\tilde{\xi }}_{1}\\ \Delta \left(\xi ,\hat{\xi }\right)-\frac{\alpha_{4}}{\varepsilon^{4}}{\tilde{\xi }}_{1} \end{array}\right]$$

where $\Delta \left(\xi ,\hat{\xi }\right)=F\left(\xi \right)-\hat{F}\left(\hat{\xi }\right)$. Defining the scaled estimation errors for each state; $\eta_{1}={\tilde{\xi }}_{1}/\varepsilon^{3}$,$\;\eta_{2}={\tilde{\xi }}_{2}/\varepsilon^{2}$, $\eta_{3}=\frac{{\tilde{\xi }}_{3}}{\varepsilon }$ and $\eta_{4}={\tilde{\xi }}_{4}$. Then the system can be written into singularity perturbed form as follows:

(42) $$\varepsilon {\dot{\eta }}_{1}=-\alpha_{1}\eta_{1}+\eta_{2}$$

(43) $$\varepsilon {\dot{\eta }}_{2}=-\alpha_{2}\eta_{1}+\eta_{3}$$

(44) $$\varepsilon {\dot{\eta }}_{3}=-\alpha_{3}\eta_{1}+\eta_{4}$$

(45) $$\varepsilon {\dot{\eta }}_{4}=-\alpha_{4}\eta_{1}+\varepsilon \Delta \left(\xi ,\hat{\xi }\right)$$

The scales estimation error can generally be denoted as: $\eta_{i}=\left(\xi_{i}-{\hat{\xi }}_{i}\right)/\varepsilon^{n-i}$ for i=1,…,4. Hence,

(46) $$\eta_{1}=\frac{\xi_{1}-{\hat{\xi }}_{1}}{\varepsilon^{3}}$$

(47) $$\eta_{2}=\frac{\xi_{2}-{\hat{\xi }}_{2}}{\varepsilon^{2}}$$

(48) $$\eta_{3}=\frac{\xi_{3}-{\hat{\xi }}_{3}}{\varepsilon }$$

(49) $$\eta_{4}=\xi_{4}-{\hat{\xi }}_{4}$$

Then by simple algebraic manipulation, the system (46)–(49) can be represented in the following form:

(50) $$\xi_{1}={\hat{\xi }}_{1}+\;\varepsilon^{3}\eta_{1}$$

(51) $$\xi_{2}={\hat{\xi }}_{2}+\;\varepsilon^{2}\eta_{2}$$

(52) $$\xi_{3}={\hat{\xi }}_{3}+\;\varepsilon \eta_{3}$$

(53) $$\xi_{4}={\hat{\xi }}_{4}+\;\eta_{4}$$

Then, (50)–(53) can be generally written as:

(54) $$\xi =\hat{\xi }+D\left(\varepsilon \right)\eta$$

where

(55) $$D\left(\varepsilon \right)=\left[\begin{array}{cccc} \varepsilon^{3} & 0 & 0 & 0\\ 0 & \varepsilon^{2} & 0 & 0\\ 0 & 0 & \varepsilon & 0\\ 0 & 0 & 0 & 1 \end{array}\right]$$

Re-arranged (54), we obtain:

(56) $$D\left(\varepsilon \right)\eta =\xi -\hat{\xi }$$

Taking derivative on both sides of (56), we obtain:

(57) $$D\left(\varepsilon \right)\dot{\eta }=\dot{\xi }-\dot{\hat{\xi }}$$

Furthermore, now substitute (21) and (39) in (57), we obtain:

(58) $$D\left(\varepsilon \right)\dot{\eta }=A\xi +B\phi \left(\xi ,u\right)-A\hat{\xi }-B\phi_{0}\left(\hat{\xi },u\right)-H\left(C\xi -C\hat{\xi }\right)$$

Re-arranged (58), we obtain:

(59) $$D\left(\varepsilon \right)\dot{\eta }=\left(A-HC\right)\left(\xi -\hat{\xi }\right)+B\left(\phi \left(\xi ,u\right)-\phi_{0}\left(\hat{\xi },u\right)\right)$$

Further, we can also write (59):

(60) $$D\left(\varepsilon \right)\dot{\eta }=\left(A-HC\right)\left(\xi -\hat{\xi }\right)+B\delta \left(\xi ,\hat{\xi }\right)$$

where $\delta \left(\xi ,\hat{\xi }\right)=\phi \left(\xi ,u\right)-\phi_{0}\left(\hat{\xi },u\right)$. Moreover, we can also write (60):

(61) $$D\left(\varepsilon \right)\dot{\eta }=\left(A-HC\right)D\left(\varepsilon \right)\eta +B\delta \left(\xi ,\gamma \left(x-D\left(\varepsilon \right)\eta \right)\right)$$

Pre multiplying $D^{-1}\left(\varepsilon \right)$ on both sides of (61), we obtain:

(62) $$\dot{\eta }=D^{-1}\left(\varepsilon \right)\left(A-HC\right)D\left(\varepsilon \right)\eta +D^{-1}\left(\varepsilon \right)B\delta \left(x,\;z,D\left(\varepsilon \right)\eta \right)$$

where

(63) $$D^{-1}\left(\varepsilon \right)=\left[\begin{array}{cccc} 1/\varepsilon^{3} & 0 & 0 & 0\\ 0 & 1/\varepsilon^{2} & 0 & 0\\ 0 & 0 & 1/\varepsilon & 0\\ 0 & 0 & 0 & 1 \end{array}\right]$$

(64) $$A-HC=\;\left[\begin{array}{cccc} 0 & 1 & 0 & 0\\ 0 & 0 & 1 & 0\\ 0 & 0 & 0 & 1\\ 0 & 0 & 0 & 0 \end{array}\right]-\left[\begin{array}{c} \alpha_{1}/\varepsilon \\ \alpha_{2}/\varepsilon^{2}\\ \alpha_{3}/\varepsilon^{3}\\ \alpha_{4}/\varepsilon^{4} \end{array}\right]\left[\begin{array}{cccc} 1 & 0 & 0 & 0 \end{array}\right]$$

Further simplifying (64) we get:

(65) $$A-HC=\left[\begin{array}{cccc} -\alpha_{1}/\varepsilon & 1 & 0 & 0\\ -\alpha_{2}/\varepsilon^{2} & 0 & 1 & 0\\ -\alpha_{3}/\varepsilon^{3} & 0 & 0 & 1\\ -\alpha_{4}/\varepsilon^{4} & 0 & 0 & 0 \end{array}\right]$$

And now (65) and (55) are used to calculate the $\left(A-HC\right)D\left(\varepsilon \right)$ as:

(66) $$\left(A-HC\right)D\left(\varepsilon \right)=\left[\begin{array}{cccc} -\alpha_{1}/\varepsilon & 1 & 0 & 0\\ -\alpha_{2}/\varepsilon^{2} & 0 & 1 & 0\\ -\alpha_{3}/\varepsilon^{3} & 0 & 0 & 1\\ -\alpha_{4}/\varepsilon^{4} & 0 & 0 & 0 \end{array}\right]\left[\begin{array}{cccc} \varepsilon^{3} & 0 & 0 & 0\\ 0 & \varepsilon^{2} & 0 & 0\\ 0 & 0 & \varepsilon & 0\\ 0 & 0 & 0 & 1 \end{array}\right]$$

(67) $$\left(A-HC\right)D\left(\varepsilon \right)=\left[\begin{array}{cccc} -\alpha_{1}\varepsilon^{2} & \varepsilon^{2} & 0 & 0\\ -\alpha_{2}\varepsilon & 0 & \varepsilon & 0\\ -\alpha_{3} & 0 & 0 & 1\\ -\alpha_{4}/\varepsilon & 0 & 0 & 0 \end{array}\right]$$

Pre multiplying (67) by $D^{-1}\left(\varepsilon \right)$ we obtain:

(68) $$D^{-1}\left(\varepsilon \right)\left(A-HC\right)D\left(\varepsilon \right)=\left[\begin{array}{cccc} 1/\varepsilon^{3} & 0 & 0 & 0\\ 0 & 1/\varepsilon^{2} & 0 & 0\\ 0 & 0 & 1/\varepsilon & 0\\ 0 & 0 & 0 & 1 \end{array}\right]\left[\begin{array}{cccc} -\alpha_{1}\varepsilon^{2} & \varepsilon^{2} & 0 & 0\\ -\alpha_{2}\varepsilon & 0 & \varepsilon & 0\\ -\alpha_{3} & 0 & 0 & 1\\ -\alpha_{4}/\varepsilon & 0 & 0 & 0 \end{array}\right]$$

Further simplifying (69) we get:

(69) $$D^{-1}\left(\varepsilon \right)\left(A-HC\right)D\left(\varepsilon \right)=\frac{1}{\varepsilon }\left[\begin{array}{cccc} -\alpha_{1} & 1 & 0 & 0\\ -\alpha_{2} & 0 & 1 & 0\\ -\alpha_{3} & 0 & 0 & 1\\ -\alpha_{4} & 0 & 0 & 0 \end{array}\right]$$

(70) $$D^{-1}\left(\varepsilon \right)\left(A-HC\right)D\left(\varepsilon \right)=\frac{1}{\varepsilon }A_{0}$$

where

(71) $$A_{0}=\left[\begin{array}{cccc} -\alpha_{1} & 1 & 0 & 0\\ -\alpha_{2} & 0 & 1 & 0\\ -\alpha_{3} & 0 & 0 & 1\\ -\alpha_{4} & 0 & 0 & 0 \end{array}\right]$$

Now to calculate the $D^{-1}\left(\varepsilon \right)B$, by using (63) we obtain as:

(72) $$D^{-1}\left(\varepsilon \right)B=\left[\begin{array}{cccc} 1/\varepsilon^{3} & 0 & 0 & 0\\ 0 & 1/\varepsilon^{2} & 0 & 0\\ 0 & 0 & 1/\varepsilon & 0\\ 0 & 0 & 0 & 1 \end{array}\right]\left[\begin{array}{c} 0\\ 0\\ 0\\ 1 \end{array}\right]=\left[\begin{array}{c} 0\\ 0\\ 0\\ 1 \end{array}\right]=B$$

Finally, substituting (70) and (72) in (62) we obtain as:

(73) $$\dot{\eta }=\frac{1}{\varepsilon }A_{0}\eta +B\delta \left(x,\;z,D\left(\varepsilon \right)\eta \right)$$

(74) $$\varepsilon \dot{\eta }=A_{0}\eta +\varepsilon B\delta \left(x,\;z,D\left(\varepsilon \right)\eta \right)$$

Since $A_{0}$ is Hurwitz, thus it is clear from the equation as the value of ε approaches zero, the uncertain term becomes zero and the error converges to zero asymptotically.

## 5. Simulation Results and Discussion

**Figure 2:**
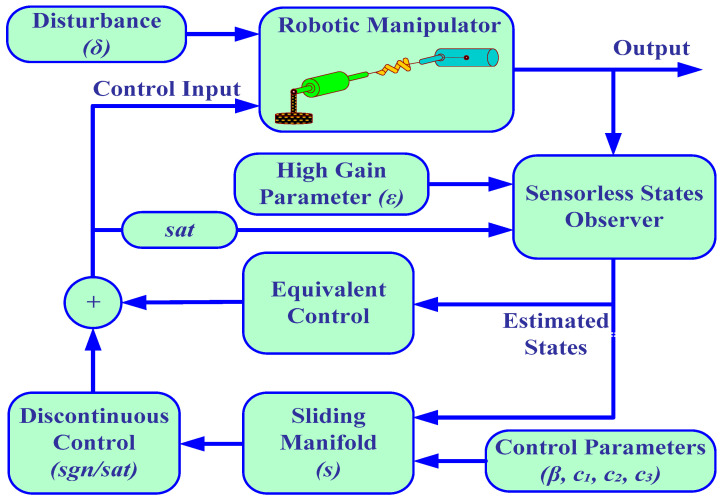
Block diagram of the SFJRM with proposed output feedback controller.

**Figure 3:**
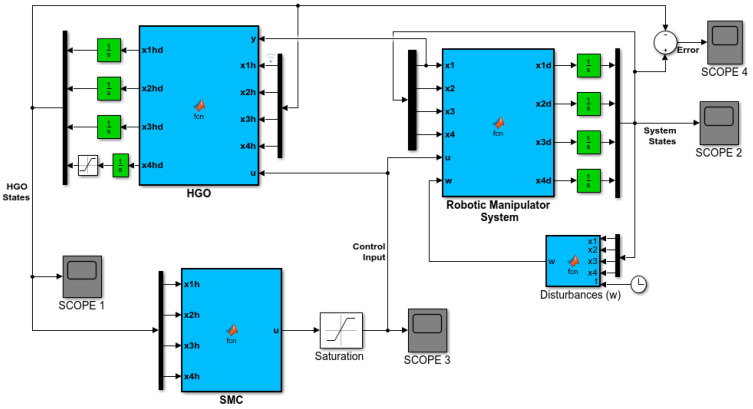
MATLAB/Simulink block diagram of the SFJRM with proposed output feedback controller in conjunction with HGO.

In this section, the sliding mode control with high-gain observer design results are validated. MATLAB/Simulink (MathWorks Inc., USA) environment is used to achieve the simulation results. The complete block diagram of the SFJRM with the proposed output feedback controller is shown in Figure 2. All the system parameters used in simulations are given in Table 1. The MATLAB/Simulink block diagram of the SFJRM with proposed output feedback controller in conjunction with HGO is shown in Figure 3. This diagram shows that the saturated control effort and system output are fed to HGO and it estimates the remaining sensorless states which are fed to controller. For assessment of observer performance, the error in observer and original states is also measured and discussed in subsequent paragraphs. The readers are directed to the supplementary files for detailed discussion on the working of model of Figure 3. The initial conditions of the system used in simulations are $x_{1}\left(0\right)=0.1$ and $x_{2}\left(0\right)=x_{3}\left(0\right)=x_{4}\left(0\right)=0$. The initial values of the high-gain observer are assumed to be ${\hat{x}}_{1}\left(0\right)={\hat{x}}_{2}\left(0\right)={\hat{x}}_{3}\left(0\right)={\hat{x}}_{4}\left(0\right)=0$. To evaluate the robustness of the output feedback under parameters variation, and external disturbances, all the parameters are varied up to ±20 percent whereas time-dependent matched disturbance is incorporated in the system model. In order to make the control globally bounded, the control input is saturated within ±100.

**Figure 4:**
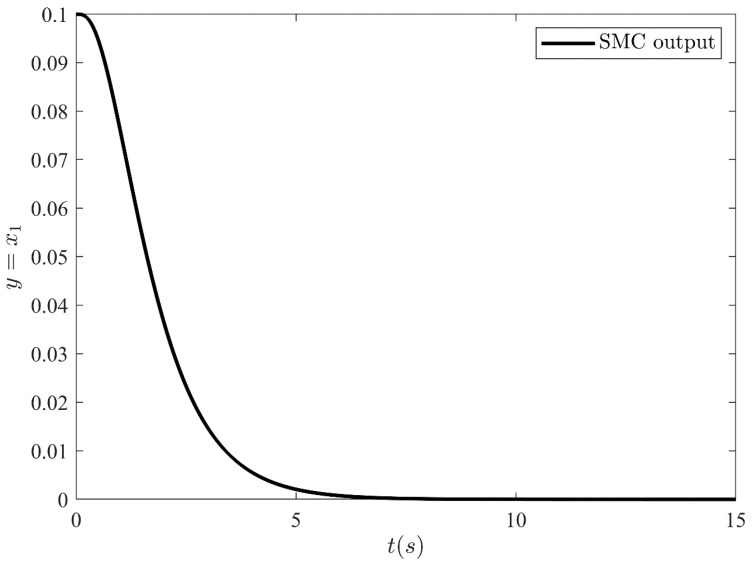
SMC output under full state feedback control.

**Figure 5:**
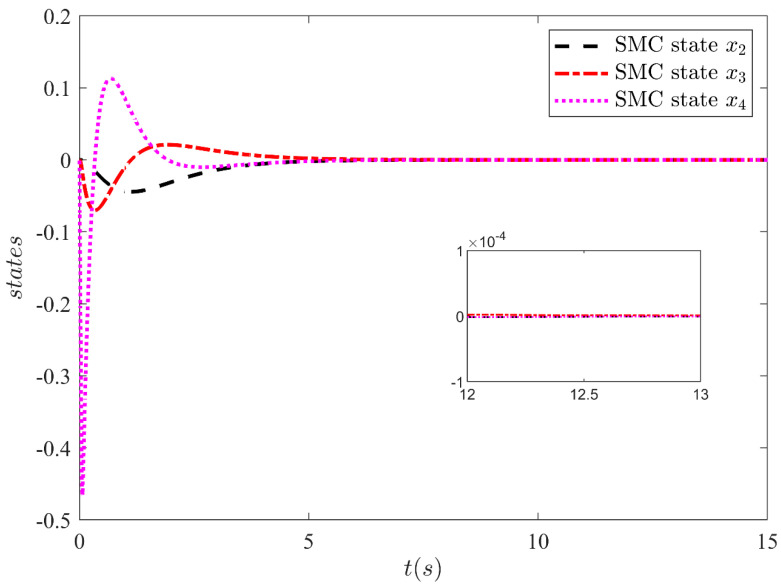
SMC state $x_{2}$,$\;x_{3}$ and $x_{4}$ under full state feedback control.

**Figure 6:**
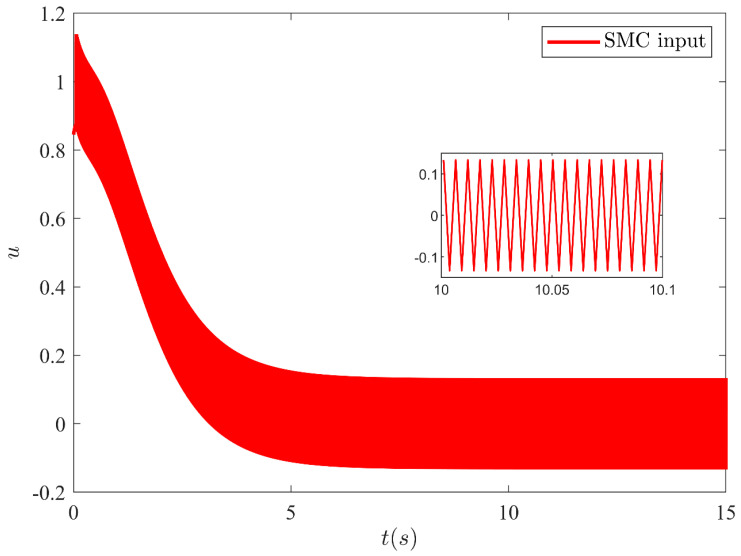
SMC control input with chattering (using signum function).

**Figure 7:**
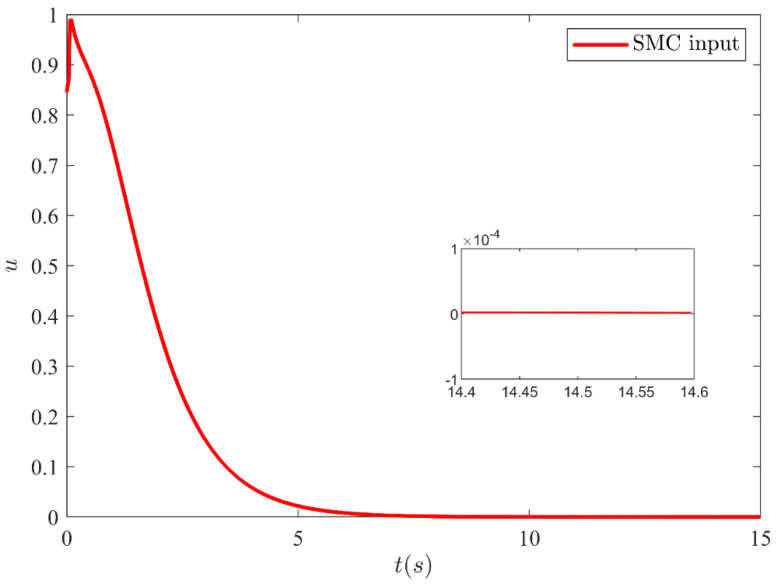
SMC control input with reduced chattering (using saturation function).

The output $y=x_{1}$ of SMC under SFC is shown in Figure 4 which is stabilized smoothly. Whereas Figure 5 illustrates the stabilization of the remaining states $x_{2}$, $x_{3}$ and $x_{4}$ under SFC. The results show that the states are perfectly stabilized after few seconds. In Table 2 output feedback control parameters are given which are used in simulations. Figure 6 shows SMC control input with the signum function, which depicts that the control input is suffering from the chattering phenomenon. To overcome the chattering, saturation is used instead of signum function due to which the control input becomes relatively smooth when states are on the sliding manifold. Figure 7 illustrates SMC control input with saturation and the reduction in chattering can be observed.

**Figure 8:**
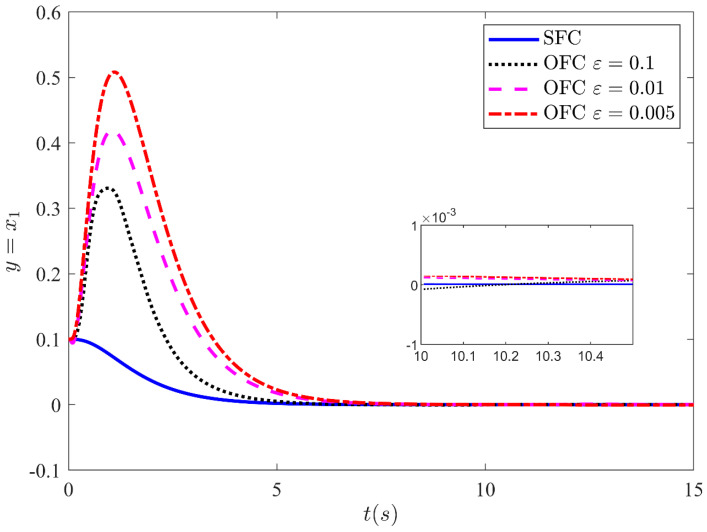
Stabilizing performance of the CL system under SFC and OFC with SMC based HGO without input saturation for three different values of *ε* = 0.1, *ε* = 0.01, and *ε* = 0.005.

Figure 8 illustrates the output $y=x_{1}$ stabilizing performance of the closed-loop (CL) system under SFC and OFC (with HGO) without input saturation. The output feedback is simulated for three different values of ε=0.1, ε=0.01, and ε=0.005. The control input is not globally bounded in this case. Peaking is induced by $\left[x_{1}\left(0\right)-{\hat{x}}_{1}\left(0\right)\right]/\varepsilon \;=0.1/\varepsilon$.

When ε is sufficiently small. The results illustrate that the response under output feedback deviates from the response under state feedback as value of high-gain parameters ε is decreased. This is unanticipated as it is expected that the response under output feedback should approach the response under state feedback as the value of ε approaches zero. This is the impact of the peaking phenomenon. Since the system and observer initial conditions are different thus peaking induces as the values of high-gain parameters are decreased. If peaking of the state takes it outside the region of attraction, it could destabilize the system.

**Figure 9:**
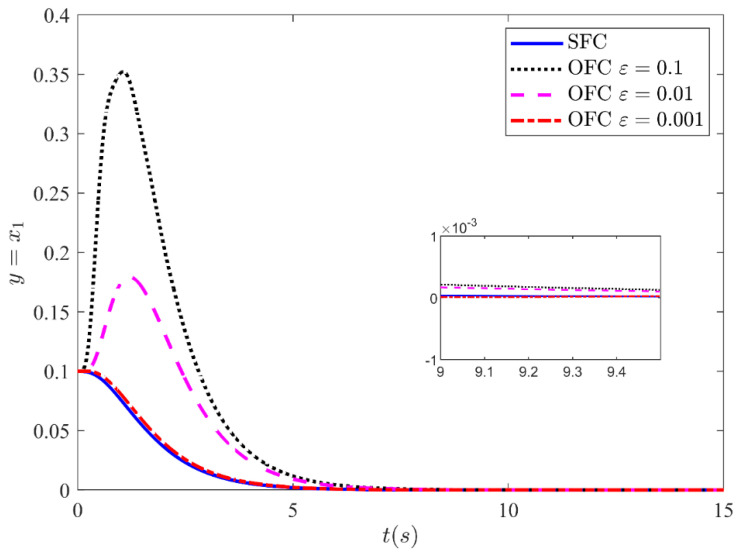
Stabilizing performance of the CL system under SFC and of OFC with SMC based HGO with input saturation for three different values of *ε* = 0.1, *ε* = 0.01, and *ε* = 0.001.

**Figure 10:**
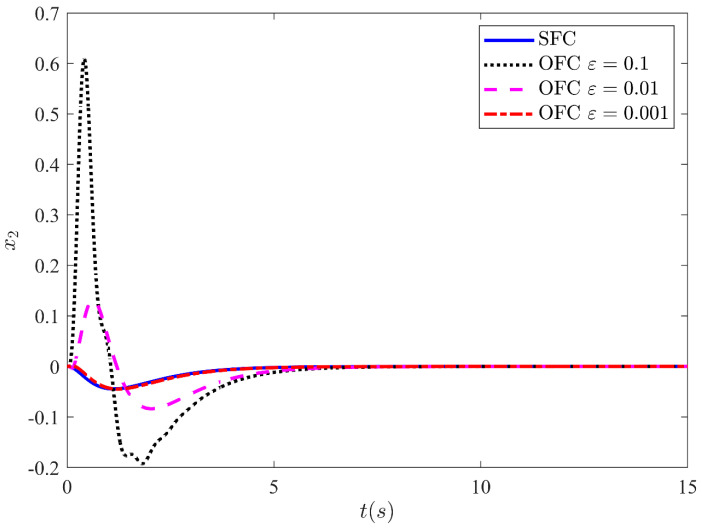
HGO based on SMC state $x_{2}$ for three different values of ε.

**Figure 11:**
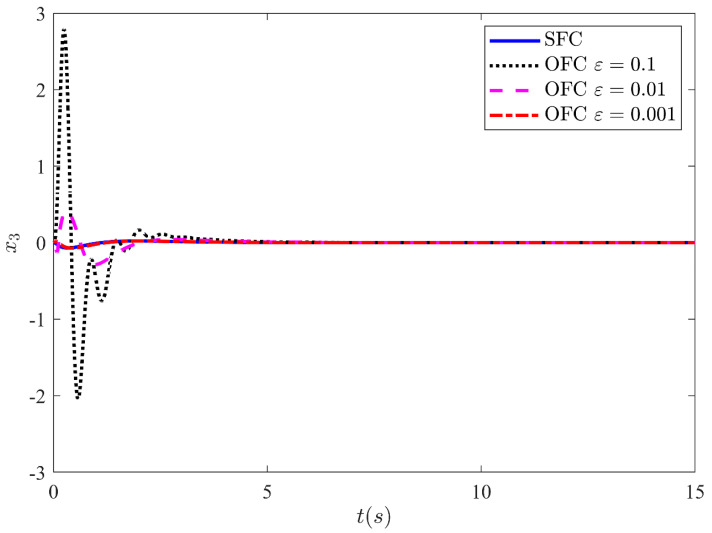
HGO based on SMC state $x_{3}$ for three different values of ε.

**Figure 12:**
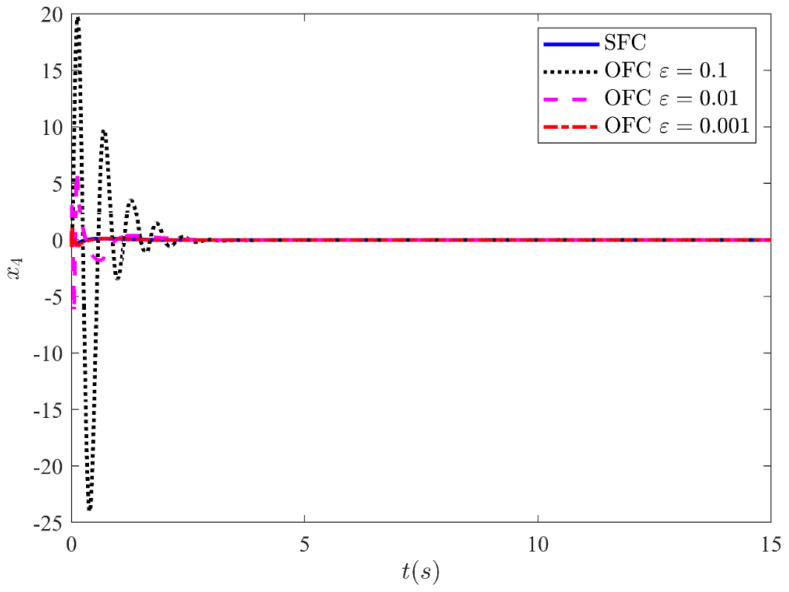
HGO based on SMC state $x_{4}$ for three different values of ε.

Fortunately, the peaking phenomenon can be overcome by the saturation of the control input outside the compact region of interest. Figure 9 shows the output $y=x_{1}$ stabilization performance of the CL system under state feedback control and output feedback control with input saturation output. The control law is made globally bounded by saturating the control input within −10 and 10. The output is shown for three different values ε=0.1, ε=0.01, and ε=0.001. The results show that the trajectories response of the CL system under output feedback approaches the trajectories response of the state feedback as the high-gain parameter values approach zero. From the results, it is noted that for a very small value of ε=0.001, the response under OFC is nearly indistinguishable from the response under SFC. This leads the performance recovery under SFC. Similarly, Figure 10, Figure 11 and Figure 12 illustrate the remaining states $x_{2}$, $x_{3}$ and $x_{4}$ response under SFC and OFC with input saturation. These states for SFC and OFC are simulated for three different values of ε=0.1, ε=0.01, and ε=0.001. Furthermore, the graph shows that the trajectories response of the CL systems under output feedback control approaches the trajectories result of state feedback control as the values of ε decreases. This shows that the HGO (with saturated input) recover the performance of state feedback control when the values of ε approaching zero.

**Figure 13:**
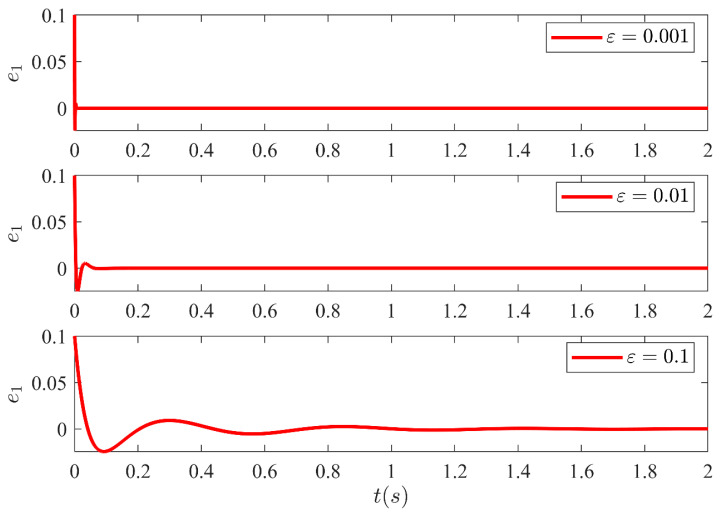
Estimation error $e_{1}$ between system state $x_{1}\;$and the estimated state ${\hat{x}}_{1}\;$of the HGO for three different values of ε.

**Figure 14:**
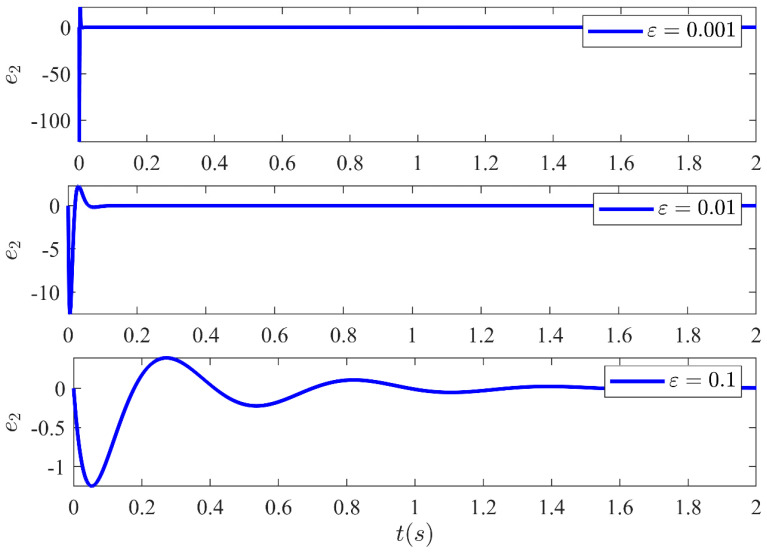
Estimation error $e_{2}$ between system state $x_{2}\;$and the estimated state ${\hat{x}}_{2}\;$of the HGO for three different values of ε.

**Figure 15:**
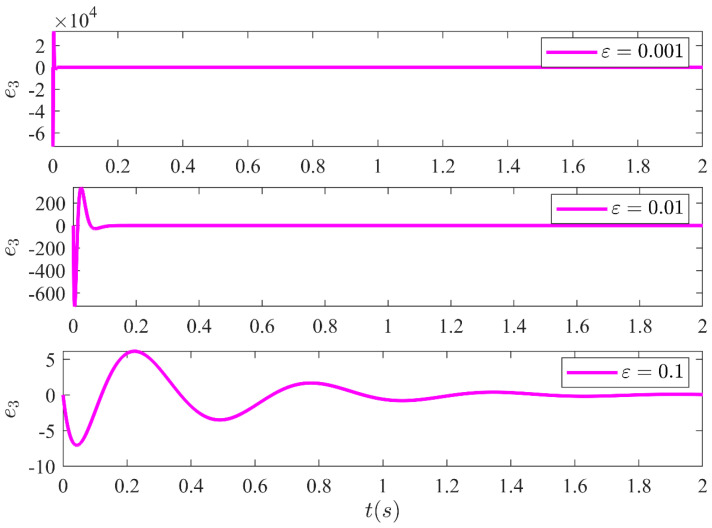
Estimation error $e_{3}$ between system state $x_{3}\;$and the estimated state ${\hat{x}}_{3}\;$of the HGO for three different values of ε.

**Figure 16:**
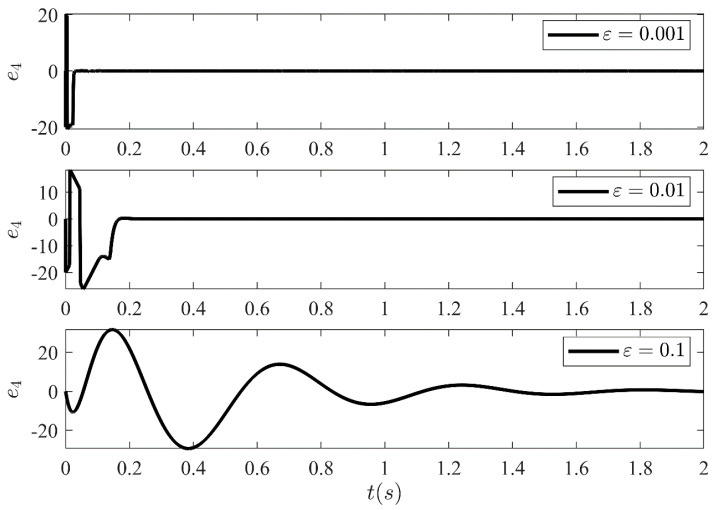
Estimation error $e_{4}$ between system state $x_{4}\;$and the estimated state ${\hat{x}}_{4}\;$of the HGO for three different values of ε.

The estimation error amongst the system states and the estimated states of the HGO are shown in Figure 13, Figure 14, Figure 15 and Figure 16 for three different values of ε. It illustrates the estimation error $e_{1}=x_{1}-{\hat{x}}_{1}$, $e_{2}=x_{2}-{\hat{x}}_{2}$,$\;e_{3}=x_{3}-{\hat{x}}_{3}$ and $e_{4}=x_{4}-{\hat{x}}_{4}$ convergence time is inversely related to ε. Moreover, it can be confirmed that once the estimated states become equal to actual states, they never leave the region of attraction for all future time. Moreover, the control technique proposed in this article is realizable and can be implemented practically without any major modification.

## 6. Conclusions

This article presents the robust OFC for a SFJRM with matched perturbations and uncertainties. A robust control technique is proposed for the semi-global stabilization problem of the angular position of the link in the SFJRM system, with the availability of only a position sensing device. In this regard, the conventional mathematical model of SFJRM is modified to a form such that the HGO and SMC can be designed for the system. The robustness property of the SMC to matched uncertainties is exploited to design a robust state feedback controller. The robustness characteristic of the HGO is used for state estimation in presence of uncertain parameters. By the virtue of the separation principle, we have designed an OFC law based on SMC and HGO in the presence of parametric uncertainties and external disturbances. The convergence analysis and numerical simulations show that the performance of the OFC approaches that of the state feedback control as the high-gain parameter is reduced. To say in nutshell, this article deals with the stabilization of SFJRM system in presence of matched perturbations and modeling uncertainties with the availability of only position sensors. The proposed methodology is supported by both theoretical analysis and simulation framework.

## Figures and Tables

**Table 1:** System parameters.

Symbol	Description	Value (Unit)
M	Mass of the link	1 kg
L	Length of the mass location from the center	1 m
k	Spring stiffness	0.3
I	Inertia of the link	$0.5\;{\mathrm{kgm}}^{2}$
J	Inertia of the actuator	$0.008\;{\mathrm{kgm}}^{2}$
g	Gravitational acceleration	$9.8\;m/s^{2}$

**Table 2:** SMC and HGO parameters.

SMC Parameter	Value	HGO Parameter	Value
$c_{1}$	6	$\alpha_{1}$	4
$c_{2}$	11	$\alpha_{2}$	6
$c_{3}$	6	$\alpha_{3}$	4
k	10	$\alpha_{4}$	1

## Supplementary Materials

The following are available online at https://www.mdpi.com/article/10.3390/s21093252/s1.
