# Supplementary material for: Robust Output Feedback Control of Single-Link Flexible-Joint Robot Manipulator with Matched Disturbances Using High Gain Observer
Source: Sensors (Basel). 2021 May 8;21(9):3252. doi: 10.3390/s21093252 (PMC8125959; doi:10.3390/s21093252)
Supplement: Supplementary file 1 [file sensors-21-03252-s001.zip › sensors-1129579-supplementary.pdf]

sensors

MDPI

Supplementary Materials

Article

Robust Output Feedback Control of Single-Link Flexible-Joint Robot Manipulator with Matched Disturbances Using High Gain Observer

Hameed Ullah *, Fahad Mumtaz Malik, Abid Raza, Naveed Mazhar, Rameez Khan, Anjum Saeed and Irfan Ahmad

Department of Electrical Engineering, CEME, National University of Sciences and Technology, Islamabad, 44000, Pakistan; malikfahadmumtaz@ceme.nust.edu.pk (F.M.M.); abid.raza@ceme.nust.edu.pk (A.R.); naveed.mazhar@ceme.nust.edu.pk (N.M.); rameez.khan@ceme.nust.edu.pk (R.K.); anjum.saeed16@ee.ceme.edu.pk (A.S.); iahmad17@ee.ceme.edu.pk (I.A.)

* Correspondence: hameed.ullah16@ee.ceme.edu.pk

Citation: Ullah, H.; Malik, F.M.; Raza, A.; Mazhar, N.; Khan, R.; Saeed, A.; Ahmad, I. Robust Output Feedback Control of Single-Link Flexible-Joint Robot Manipulator with Matched Disturbances Using High Gain Observer. *Sensors* **2021**, *21*, 3252. https://doi.org/10.3390/s21093252

Received: 14 February 2021
Accepted: 2 April 2021
Published: 8 May 2021

Publisher's Note: MDPI stays neutral with regard to jurisdictional claims in published maps and institutional affiliations.

Copyright: © 2021 by the authors. Licensee MDPI, Basel, Switzerland. This article is an open access article distributed under the terms and conditions of the Creative Commons Attribution (CC BY) license (http://creativecommons.org/licenses/by/4.0/).

1. Overview of MATLAB/Simulink Model.

In this file we have explained the MATLAB/Simulink Model, its inside coding, and the main results.

Explanation: The following is the main MATLAB/Simulink Model, which consist of the robotic manipulator system block, high gain observer (HGO) block, and sliding mode control (SMC) block. The inside working of all these blocks is explained in the next section. In the Simulink block the system states $x_1, x_2, x_3,$ and x_4 are represented by x1, x2, x3 and x4, respectively, whereas the derivative of the system states i.e., $\dot{x}_1, \dot{x}_2, \dot{x}_3,$ and $\dot{x}_4$ are represented by x1d, x2d, x3d and x4d, respectively. The output of the system $(y = x_1)$ is fed into the HGO block. On the other hand, in the Simulink block of the HGO, the HGO states $\hat{x}_1, \hat{x}_2, \hat{x}_3,$ and $\hat{x}_4$ are represented by x1h, x2h, x3h and x4h, respectively, whereas the derivative of the HGO states i.e., $\dot{\hat{x}}_1, \dot{\hat{x}}_2, \dot{\hat{x}}_3,$ and $\dot{\hat{x}}_4$ are represented by x1hd, x2hd, x3hd and x4hd, respectively. The HGO states are then fed into SMC block. The output of the SMC acts as the input (u) of the system and it is also fed into HGO block. Disturbance is defined outside the system block, denoted by "Disturbance (w)" block and it feeds the disturbance w into the system block. The SCOPE-1 represents the estimated states or HGO states; SCOPE-2 represents the original states or system states, SCOPE-3 represents control input designed in SMC block, SCOPE-4 represents the error (the difference between the system states and HGO states). These simulations are carried out in Simulink, MATLAB 2018a.

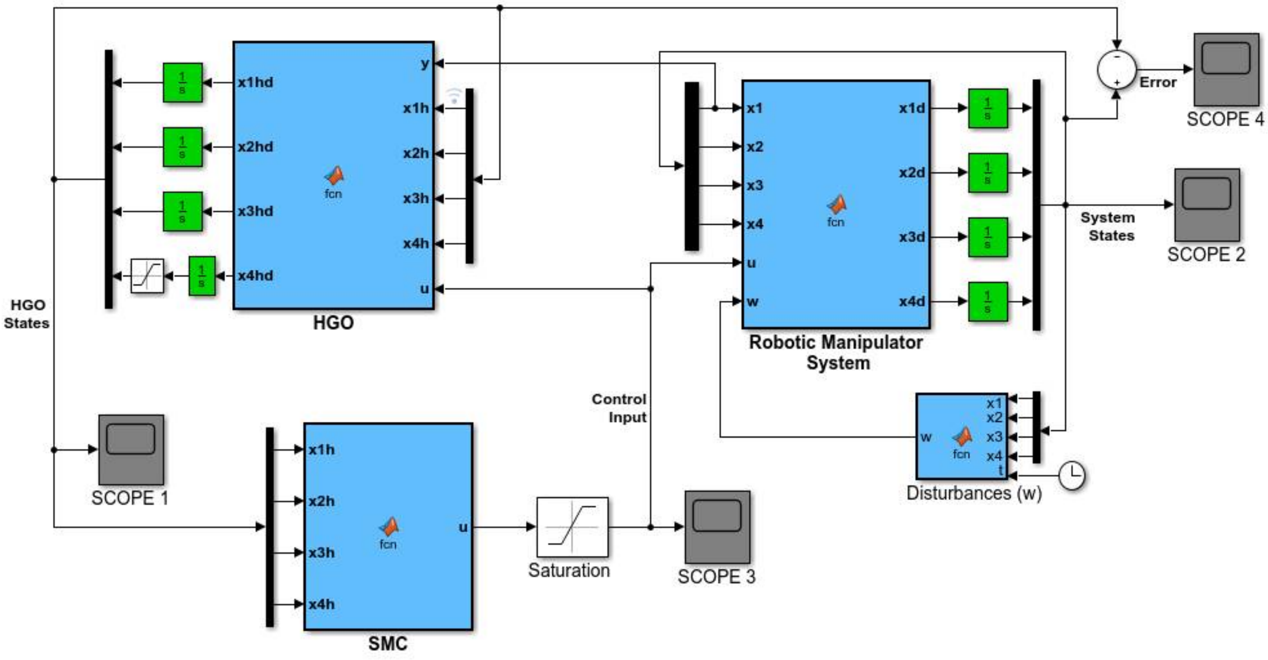

Figure 3. MATLAB/Simulink block diagram of the Single-link Flexible-Joint Robot Manipulator with proposed output feedback controller (SMC) in conjunction with the high gain observer (HGO).

2. Code:

In this section all the code inside the system block, the HGO block, SMC block and disturbances block are written as the following:

a. Code inside System block:

```
function [x1d,x2d,x3d,x4d] = fcn(t,x1,x2,x3,x4,u,w)
% Original System parameters (Perturbed).
M  = 1;
L  = 1;
I  = 0.5;
k  = 0.3;
J  = 0.008;
g  = 9.8;
a  = M*g*L/I;
b  = k/I;
c  = k/J;
d  = 1/J;
F  = -(a*cos(x1)+b+c)*x3 + a*(x2^2-c)*sin(x1);
g  = b*d;
x1d = x2;
x2d = x3;
x3d = x4;
x4d = F + g*u + w;
```

b. Code inside HGO block:

```
function [x1hd,x2hd,x3hd,x4hd] = fcn(y,x1h,x2h,x3h,x4h,u)
% Original System parameters (Perturbed).
% M  = 1;
% L  = 1;
% I  = 0.5;
% k  = 0.3;
% J  = 0.008;
% g  = 9.8;
% Nominal Parameters used in control/observer design.
M  = 0.8;
L  = 1.25;
I  = 0.5;
k  = 0.27;
J  = 0.0072;
g  = 9.8;
a  = M*g*L/I;
b  = k/I;
c  = k/J;
d  = 1/J;
F   = -(a*cos(x1h)+b+c)*x3h + a*(x2h^2-c)*sin(x1h);
g   = b*d;
%HGO characteristic polynomial nominal constant
a1  = 4.5;
a2  = 6.5;
a3  = 4.5;
a4  = 1.2;
%varying epsilon (e) for different values (Figure 9-16)
e  =0.001;
% e =0.01;
% e =0.1;
%HGO Gains
h1  = a1/e;
h2  = a2/e^2;
h3  = a3/e^3;
h4  = a4/e^4;
 %HGO states
x1hd= x2h + h1*(y-x1h);
x2hd= x3h + h2*(y-x1h);
x3hd= x4h + h3*(y-x1h);
x4hd= F + g*u + h4*(y-x1h);
```

c. Code inside SMC block:

```
function u = fcn(x1,x2,x3,x4)
% Original System parameters (Perturbed).
% M  = 1;
% L  = 1;
% I  = 0.5;
% k  = 0.3;
% J  = 0.008;
% g  = 9.8;
% Nominal Parameters used in control/observer design.
M  = 0.8;
L  = 1.25;
I  = 0.5;
k  = 0.27;
J  = 0.0072;
g  = 9.8;
a  = M*g*L/I;
b  = k/I;
c  = k/J;
d  = 1/J;
F  = -(a*cos(x1)+b+c)*x3 + a*(x2^2-c)*sin(x1);
g   = b*d;
%SMC characteristic polynomial nominal constant.
a1=6.5;
a2=11.5;
a3=6.5;
s=a1*x1+a2*x2+a3*x3+x4;
k=12.5;
%k=10;
e=0.1;
% v=sign(s);              %signum function for Figure 4-6.
%% Saturation Function for reduced chattering (Figure 7-16)
if abs(s)> e
        v=sign(s);
else
        v = (s/e);
end
 u  = (1/g)*(-a1*x2-a2*x3-a3*x4-F-k*v);
```

d. Code inside Disturbance block:

```
function w =fcn(x1,x2,x3,x4,t)
w1=0.05*sin(t);          % External disturbance (non-vanishing)
w2=0.5*sin(x2*x3*t);     % Mixed disturbance Term(vanishing)
w=w1+w2;
```

3. The Steps to Extract the Article Results from attached simulations:

The simulation results of this article are provided in Figures 4-16. All these results are produced by some alteration in the attached simulation ((MatlabSimulink_Block_Diagram_of_SFJFM_SMC_HGO.Slx), produced in MATLAB 2018a). These alterations are given below:

a. Figure 4, Figure 5 and Figure 6 show the System output, states, and input with sliding mode control (SMC) under full state feedback control, respectively. These figures can be reproduced by evading the high gain observer (HGO) block and connecting the system states direct to SMC as feedback. Moreover, signum function is used in SMC code (see "2c. Code inside SMC block" in this file). Scope 2 and Scope 3 show the graphs of states and input, respectively.
b. Figure 7 shows SMC control input with reduced chattering. This figure can be reproduced by evading only HGO block and connecting the system states direct to SMC as feedback. The saturation function is used instead of signum function to reduce the chattering (see "2c. Code inside SMC block" in this file).
c. Figure 8 shows the Stabilizing performance of the closed loop (CL) system under state feedback controller (SFC) and output feedback control (OFC) with SMC-based HGO without input saturation for three different values of ε. In this case the same Simulink model is used (which is attached with this file) except the saturation block before HGO is removed.
d. Figure 9 to Figure 12 show system states stabilizing performance of the CL system under SFC and of OFC with SMC-based HGO with input saturation for three different values of ε. This result can be achieved from the Simulink block diagram attached with this file, which shows all the system states under SMC in conjunction with HGO. These graphs can be produced by attached simulation via scope 4.
e. Figure 13 to Figure 16 show the estimation error $\boldsymbol{e}$ between system state $\boldsymbol{x}$ and the estimated state $\hat{x}$ of the HGO for three different values of $\boldsymbol{\varepsilon}$. These graphs can be produced by attached simulation via scope 4.
